# Sustainable wastewater decontamination from chlortetracycline using kaolin–alginate beads: adsorption mechanisms and practical applications

**DOI:** 10.1098/rsos.250439

**Published:** 2025-10-08

**Authors:** Wei Liu, Meriem Fizir, Sami Touil, Amina Richa, Douba Houda, Pinping Wu, Jiang Qian, Yongbing Zhang, Yulong Wang, Jing Ding

**Affiliations:** ^1^ Zhejiang Pharmaceutical University, Ningbo, Zhejiang 315500, People's Republic of China; ^2^ Laboratory of Precision Agriculture, Environment and Sustainable Development, Khemis Miliana University, Khemis Miliana 44225, Algeria; ^3^ Laboratoire de Valorisation des Substances Naturelles, Khemis Miliana University, Khemis Miliana 44225, Algeria; ^4^ Key Laboratory for Core Technology of Generic Drug Evaluation National Medical Product Administration, Hangzhou 310052, People's Republic of China

**Keywords:** kaolin, alginate, activated carbon, chlortetracycline, adsorption

## Abstract

Addressing the need for cost-effective alternatives to activated carbon (AC) for chlortetracycline (CTC) removal, this study developed sustainable kaolin–alginate composite beads (KN@Alg). The adsorption performance of KN@Alg was systematically evaluated compared with pristine KN and AC through kinetics, isotherms and thermodynamics. Regeneration cycles and X-ray photoelectron spectroscopy analysis were employed to assess reusability and elucidate mechanisms. Results demonstrated that incorporating of KN into the alginate matrix significantly enhanced the adsorption capacity to 68.74 mg g^−1^, surpassing that of KN (42.76 mg g^−1^) and approaching that of AC (102.96 mg g^−1^). KN@Alg achieved 93.7% removal efficiency in dynamic experiments, demonstrating practical applicability. Thermodynamics confirmed a spontaneous and exothermic process. Mechanistic studies revealed that CTC uptake onto KN@Alg involves multifunctional mechanisms, including n–π interactions, hydrogen bonding, electrostatic attraction, cation exchange and calcium ion-bridging. Notably, KN@Alg exhibited superior renderability, retaining approximately 76% efficiency after four cycles, outperforming both AC and KN. Compared with the high cost of AC, KN@Alg integrates the rigid framework of KN with the functional advantages of alginate, addressing the limitations of low adsorption capacity and instability of pure components while achieving comparable removal efficacy. These findings highlight KN@Alg as a sustainable, cost-effective alternative for CTC-contaminated water treatment.

## 1. Introduction

The widespread presence of residual antibiotics in environment systems has become a critical global issue, garnering significant scientific and public attention [1]. Chlortetracycline (CTC), a widely used tetracycline antibiotic in human therapy and livestock breeding, poses serious environmental and health risks if discharged without proper treatment [2,3]. Generally, some antibiotic compounds exhibit strong hydrophilicity, with substantial evidence confirming their penetration into groundwater sources and drinking water [4]. The potential adverse effects of their biological toxicity and the low removal rates of antibiotics by traditional wastewater treatment processes have intensified public concerns about health risks. Thus, finding effective CTC removal methods is crucial for environmental and human health.

Advanced technologies like biodegradation, membrane separation and chemical oxidation have been developed for CTC remediation [5–7]. While effective, these methods face high costs, infrastructure needs and skilled personnel requirements, limiting their widespread use [8]. Among these, adsorption is a superior method bceause of its high efficiency, low cost and minimal secondary pollution [3]. Numerous materials, such as gels [9], organic polymers [10], metal–organic frameworks [11], carbon- or activated carbon (AC)-based materials [12,13] and boron nitride [14], have been used as CTC adsorbents. Generally, AC, the most common, is limited by high production and regeneration costs. Thus, developing cost-effective and scalable adsorbents is crucial.

Recently, clays have gained attention for their abundance, cost-effectiveness and environmental friendliness [15]. Kaolin (KN), natural clay, is particularly promising due to its global availability, low cost and unique structure [16]. These inherent properties make clay-based materials highly attractive and sustainable candidates for adsorption applications [17]. KN is a hydrous aluminium phyllosilicate with each layer consisting of an Al–OH octahedral sheet called the ‘aluminon surface’ and a Si–O tetrahedral sheet called the ‘siloxane surface’. These two sheets share the top O atom [16]. The predominantly negative and hydrophobic surface charge observed originates from the presence of siloxane groups, while the surface of aluminium alcohol is hydrophilic and positively charged. Therefore, the various layers of KN are connected together through hydrogen bonding and dipole interaction. There is a phenomenon of isomorphic substitution of Si^4+^ by Al^3+^ and Fe^3+^, as well as Al³^+^ by Mg^2+^, in the crystal lattice of KN, resulting in the generation of a small amount of permanent negative charge on the (001) crystal plane. The edges of KN crystals contain exposed oxygen (O) atoms and hydroxyl (OH) groups, which can undergo pH-dependent protonation/deprotonation, resulting in a variable surface charge. The amount of this variable charge fluctuates in accordance with the pH level of the surrounding medium, making these groups active adsorption sites [18]. Moreover, the high specific surface area and the unique composition of a hydroxyl group on the surface enable it to interact with various substances. KN has demonstrated a high removal rate for CTC [19]. However, KN faces challenges like pressure drop during filtration and difficulty in recovery, limiting its practical use. To address these issues, researchers have turned to biopolymer composite beads. Sodium alginate (SA), a biocompatible and biodegradable biopolysaccharide, is commonly used, primarily sourced from brown algae of the *Phaeophyceae* family and it stands out as a versatile and eco-friendly candidate due to its ability to form functional complexes with cations [20]. SA crosslinks with Ca²^+^ to form insoluble ‘egg-box’ structures, creating easily separable beads [21]. Despite this advantage, the limited reusability of SA beads poses a significant challenge for practical applications.

To enhance the stability of SA, researchers have combined it with materials like graphene oxide [22], double network polyvinyl alcohol [23] and Ga-based metal organic gel [24] for tetracycline antibiotic adsorption. However, these composites are costly, require complex synthesis, and lack standardized protocols, limiting their environmental remediation potential [25]. Clay minerals, such as KN and montmorillonite (Mt), offer a promising alternative due to their layered structure, which can enhance the mechanical strength and stability of alginate beads during regeneration, making them suitable for practical use [26]. For example, Mt modified with Alg has been used as an adsorbent for tetracycline antibiotics, exhibiting maximum adsorption capacities of 745 mg g^−1^ at a concentration of 4000 mg l^−1^ and less than 100 mg g^−1^ at concentrations below 100 mg l^−1^ [25]. Despite these advancements, composite beads of SA with KN have not been extensively studied for CTC adsorption. KN was selected in this study due to its stability, cost-effectiveness and availability. Unlike Mt, KN is less prone to expansion and maintains structural integrity during adsorption, which is crucial for effective performance. Additionally, KN is more affordable and easier to obtain than Mt, making it a preferred adsorbent for scalable and practical applications [27].

This study explores the use of cost-effective calcium crosslinked alginate–kaolin beads for CTC adsorption from water. The performance of KN@Alg was evaluated in both batch and dynamic systems, with comparisons with AC and pristine KN conducted exclusively in the batch system. Furthermore, the interaction mechanism between CTC and KN@Alg was investigated, a novel approach that has not been previously explored. Comparing KN@Alg with AC is critical because AC is a benchmark in water treatment due to its high contaminant removal efficiency. If KN@Alg demonstrates comparable or superior performance at a lower cost, it could serve as a sustainable and economical alternative. Moreover, KN@Alg, derived from natural materials, offers an eco-friendly solution. Analysing the adsorption mechanisms of KN@Alg versus AC can advance water treatment technologies, particularly for pharmaceutical contaminants like CTC. The key objectives of this study are: (i) synthesizing and characterizing KN@Alg composite material, (ii) systematically evaluating CTC adsorption on AC, KN and KN@Alg under varying conditions and (iii) investigating adsorption kinetics, equilibrium and thermodynamics to elucidate adsorption behaviours and mechanisms. Additionally, this study aims to apply KN@Alg to real water samples using a dynamic system for CTC removal, providing practical insights for scaling up its use in environmental remediation.

## 2. Material and methods

### 2.1. Chemicals and reagents

SA (NaC_6_H_7_O_6_, AR, viscosity 1.05−1.15 Pa s) was bought from Dengfeng Chemical Reagent Factory (Tianjin, China). Sodium hydroxide (NaOH, AR), hydrochloric acid (HCl, AR) and calcium chloride (CaCl_2_, 94%, 1−3 mm) were purchased from Shanghai Macklin Biochemical Co. Ltd (China). CTC (≥91% dry basis, HPLC) with the chemical formula ‘C_22_H_23_ClN_2_O_8_’ and a molecular mass of 478.88 g mol^−1^ was acquired from Shanghai Aladdin Biochemical Technology Co. Ltd (China). KN (AR) clay from Biochem Chemopharma (France) was used as received. AC (untreated, granular, ≤5 mm) was acquired from Sigma-Aldrich (China).

### 2.2. Synthesis of alginate composite beads based on kaolin

**Figure 1.**
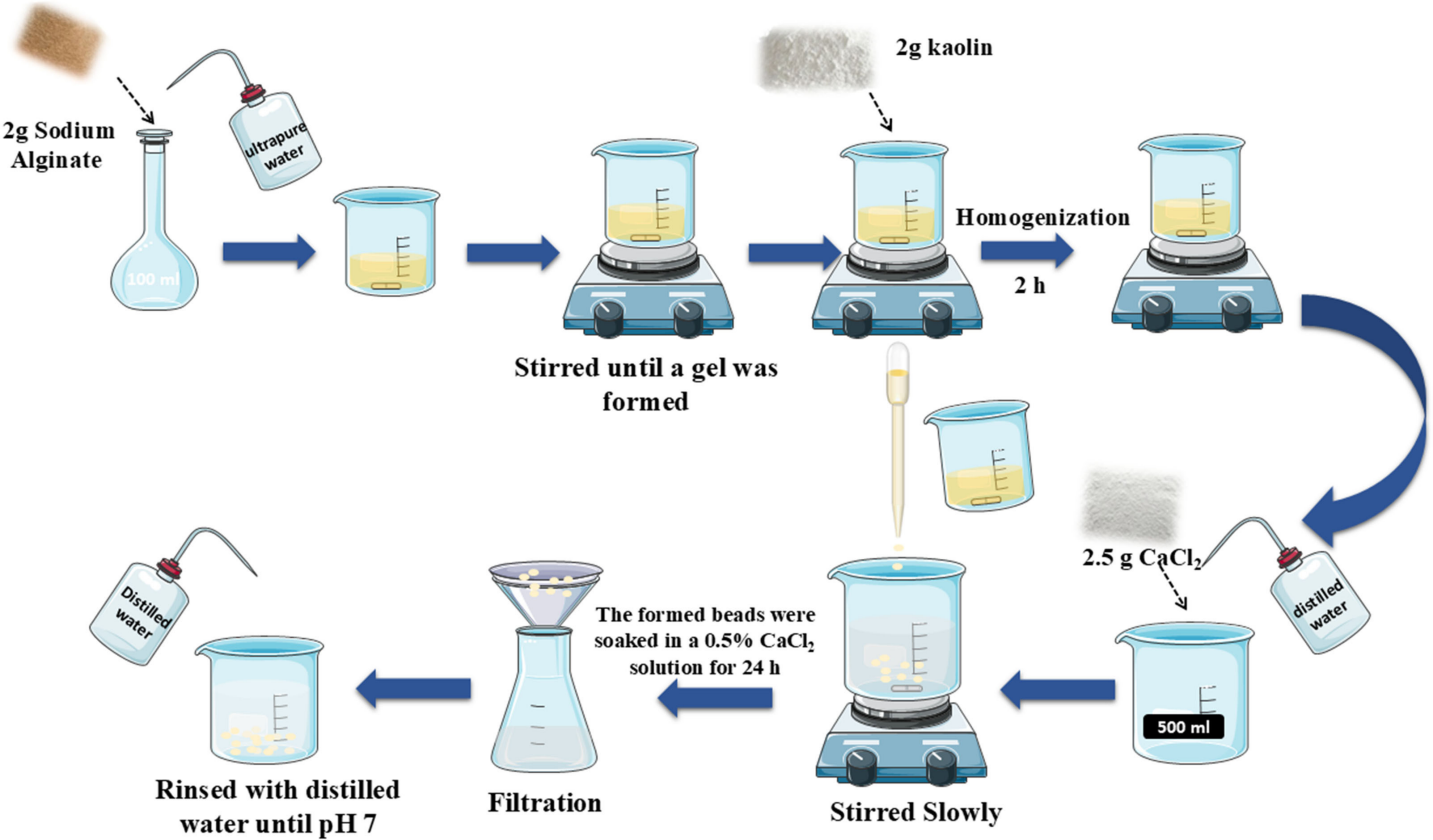
Synthesis procedures of calcium crosslinked KN@Alg beads.

The KN@Alg composite beads were fabricated through an extrusion-based synthesis approach [25]. Initially, SA (2 g) was dispersed in distilled water (100 ml) under stirring over a 2 h period. Next, 2 g of KN was systematically introduced into the colloidal SA matrix, followed by thorough mixing to obtain a homogeneous SA–KN suspension. This mixture was then dropped into a 0.5% CaCl_2_ solution with a dropper (figure 1). The calcium ions caused the SA–KN mixture to crosslink immediately upon contact with the calcium solution, forming beads. After solidification, the beads were thoroughly rinsed with distilled water to remove any excess CaCl_2_. The absence of chloride ions in KN@Alg beads was verified by AgNO_3_ solution, and the final beads were kept in distilled water for subsequent experimental applications [28].

### 2.3. Characterization

The surface characteristics of KN and KN@Alg were investigated employing scanning electron microscopy (SEM, Regulus 8100, Hitachi, Japan and Quattro E, Thermo Scientific™, USA, respectively). Energy-dispersive spectroscopy (EDS, Gemini SEM 5000, ZEISS, Germany) was utilized for the elemental changes before and after the reaction between KN@Alg beads and CTC. Fourier transform infrared (FTIR, FTIR-8400 spectrometer, Shimadzu, Japan) was applied to record FTIR spectra of KN and KN@Alg both prior to and after CTC adsorption. Samples were prepared as KBr pellets under infrared lamp drying to prevent moisture interference from ambient air, and spectra were recorded over the wavenumber range of 4000−400 cm⁻¹. The phase structures of clay and KN@Alg were determined by powder X-ray diffraction (XRD, D8 ADVANCE, Bruker, Germany) with Cu Kα radiation in the range of 5−90° (2*θ*). X-ray photoelectron spectroscopy (XPS) measurements were performed using a Thermo Scientific™ ESCALAB 250Xi spectrometer (Thermo Fisher Scientific, USA) to analyse surface elemental composition. All spectra were acquired at ambient temperature under ultra-high vacuum (5 × 10⁻⁹ Pa) using monochromatic Al Kα radiation (*hν* = 1486.6 eV), with an electron take-off angle of 30° (defined as the angle between the sample surface and analyser entrance). Binding energies were calibrated against the adventitious carbon C 1s peak, typically referenced at 284.6 or 284.8 eV, to correct for charge effects.

### 2.4. Batch adsorption studies

#### 2.4.1. Parametric evaluation of chlortetracycline removal by activated carbon, kaolin and KN@Alg

The effectiveness of AC, KN and KN@Alg for CTC removal was assessed through batch adsorption experiments. Various parameters, including the adsorbent dosage (0.4−4 g l^−1^), solution pH value (2–12), temperature (298–328 K), adsorption time (5 min to 7 h), and initial CTC concentration (20–100 mg l^−1^) [28], were analysed to identify the optimal adsorption capacity with 10 ml of CTC solutions. The pH of the solutions was adjusted using diluted acids or bases [13]. Subsequently, the mixtures were shaken at a fixed temperature and a speed of 200 r.p.m. for predetermined contact duration. Following the attainment of adsorption equilibrium, the absorbance of the supernatant after centrifugation was measured at 365 nm to determine the concentration of CTC. The adsorption capacity (mg g^−1^) and removal efficiency (%) of the adsorbents were calculated using equations (2.1)–(2.2):


(2.1) $$Q=\frac{\left(C_{0}-C_{e}\right)\times V}{m},$$

(2.2) $$Removal\;efficiency\;\left(\%\right)=\frac{\left(C_{0}-C_{e}\right)}{C_{0}}\times 100,$$

where *C*_0_ and *C_e_* (mg l^−1^) were the initial and equilibrium concentrations of unabsorbed CTC in the solution, respectively, *V* (ml) denoted solution volume and *m* (g) signified the amount of adsorbent used.

#### 2.4.2. Zero charge point profiling of adsorbents

The zero charge point (pH_pzc_) values of three adsorbents were determined by NaCl addition[29]. 0.5 g of AC, KN or KN@Alg was mixed with 20 ml of 0.01 M NaCl after adjusting the pH value of the solution and then the beaker was stirred at 298 K and 160 r.p.m. for 24 h. The final pH value in the supernatant of each beaker was measured after centrifuging. The pH_pzc_ values of studied adsorbents (AC, KN and KN@Alg beads) were determined from the pH_initial_ versus pH_final_–pH_initial_ plot [30].

#### 2.4.3. Kinetic and isotherm experiments

For the kinetic experiments, 0.8 g l^−1^ of AC and 4 g l^−1^ of KN and KN@Alg were separately introduced into CTC solutions with an initial concentration of 100 mg l^−1^ at a pH value of 6. The solutions were shaken at 298 K for different reaction durations ranging from 5 min to 7 h. To analyse the CTC adsorption kinetics on the selected adsorbents, the pseudo-first-order kinetic (PFO), pseudo-second-order kinetic (PSO), and Elovich models were employed to determine the rate constants of the adsorption process. Additionally, to evaluate the impact of diffusion on the adsorption, an intraparticle diffusion (IPD) model was used to fit the kinetic data [24]. The nonlinear equations for these models are presented in electronic supplementary material, table S1 [31].

To evaluate the adsorption isotherms of these adsorbents, 0.8 g l^−1^ of AC and 4 g l^−1^ of KN and KN@Alg were separately mixed with CTC solutions at concentrations from 10 to 100 mg l^−1^, maintaining the pH at 6 and the temperature at 298 K. The adsorption equilibrium of CTC onto the adsorbents was analysed using Langmuir, Freundlich and Temkin models. The nonlinear equations for these isotherm models are provided in electronic supplementary material, table S2 [32]. The separation factor *R_L_* in the Langmuir model reflects the strength of interaction between adsorbate and adsorbent, which can be calculated by equation (2.3). When 0 < *R_L_* < 1, the adsorption process is thermodynamically favourable, indicating spontaneous adsorption [33].


(2.3) $$R_{L}=\frac{1}{1+C_{0}K_{L}}.$$

#### 2.4.4. Thermodynamic studies

To elucidate the mechanism of temperature-dependent adsorption behaviour between the adsorbent and CTC, key thermodynamic parameters (Δ*G°*, Δ*H°* and Δ*S°*) were calculated using the Van’t Hoff equation [34,35]:


(2.4) $$K=\frac{Q_{e}}{C_{e}},$$

(2.5) $$\Delta G^{\circ }=-RT\ln K,$$

(2.6) $$\ln K=\left(\frac{\Delta S^{\circ }}{R}\right)-\left(\frac{\Delta H^{\circ }}{R}\right)\frac{1}{T},$$

(2.7) $$\Delta G^{\circ }=\Delta H^{\circ }-T\Delta S^{\circ },$$

where *K* and *R* are the equilibrium constant and ideal gas constant, respectively.

#### 2.4.5. Reusability study

The robustness and economic viability of an adsorbent are vital characteristics for evaluating its effectiveness. A study on adsorbent regeneration was conducted under optimal experimental conditions. Specific dosages of KN, KN@Alg and AC were used in the water sample to adsorb CTC over four cycles. The adsorbent was regenerated with 20 ml of a solution containing 0.1 M HCl, 0.1 M NaCl and distilled water for 1 h [28]. Subsequently, the materials were separated, thoroughly washed with distilled water and prepared for subsequent cycles.

#### 2.4.6. Solid-phase extraction cartridge and continuous bead column experiments

To assess the potential for practical applications, experiments were conducted using both solid-phase extraction (SPE) cartridges and continuous bead columns. For the SPE cartridge experiments, 40 beads were loaded into a 6 ml empty SPE cartridge (6.7 cm height). A 5 mg l^−1^ CTC solution was prepared in three aqueous matrices—distilled water, tap water and river water (collected from Ningbo, China)—and introduced into the SPE columns via top-loading at a controlled flow rate of 1 drop s^−1^. The effluent concentration was measured to determine the removal rate. In the continuous-flow bead column experiment, a glass column (1.5 cm internal diameter × 15 cm height) was packed with a 1 cm quartz sand layer at the base. A 5 mg l^−1^ CTC-contaminated river water solution was continuously pumped through the column at 1 ml min^−1^ in a top-to-bottom direction using a peristaltic pump [9].

## 3. Results and discussion

### 3.1. Characterization

**Figure 2.**
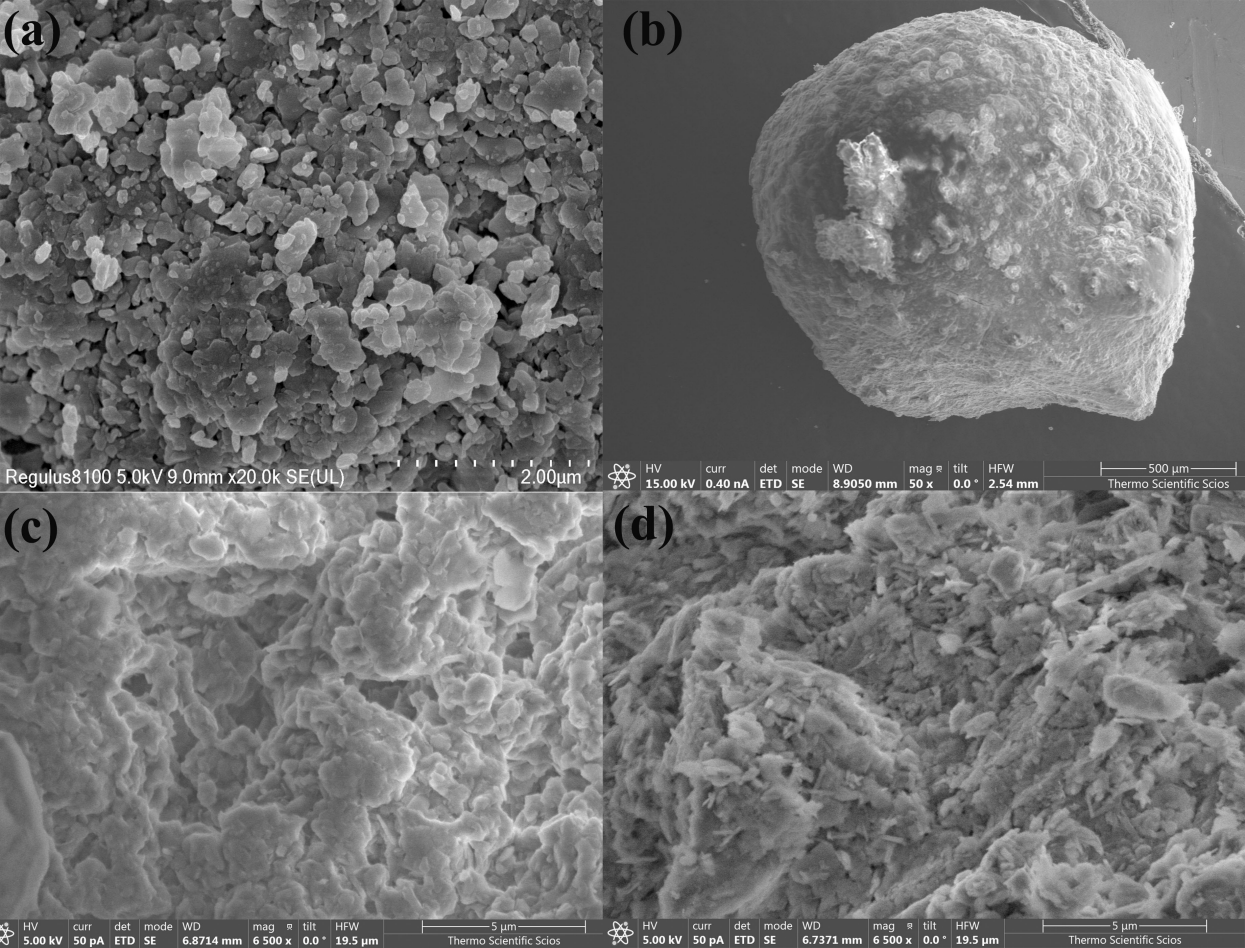
SEM images of KN (a), KN@Alg (b,c) and KN@Alg@CTC (d).

The surface morphology of KN and KN@Alg beads was investigated through SEM. As depicted in figure 2, KN particles exhibit angular crystalline formations with irregular contours, displaying dimensions averaging 0.2 mm [28]. By contrast, the SEM images of KN@Alg composite beads, which have a diameter of approximately 1 mm (figure 2b,c), reveal an irregular surface featuring cracks, undulations, folds and pores resulting from the incorporation of KN clay into the polymer matrix [36]. The KN@Alg beads exhibited an irregular microstructure composed of polyhedral particles of varying sizes, consistent with observations reported in previous studies [37]. After CTC adsorption, SEM analysis of the composite beads (figure 2d) revealed that the pleated voids on the bead surfaces were filled with a substantial number of fine particles. These particles are likely formed due to the flocculation of CTC molecules, resulting from local supersaturation on the bead surface. Similar morphological transformations following adsorption have been reported in previous studies [38,39].

**Figure 3.**
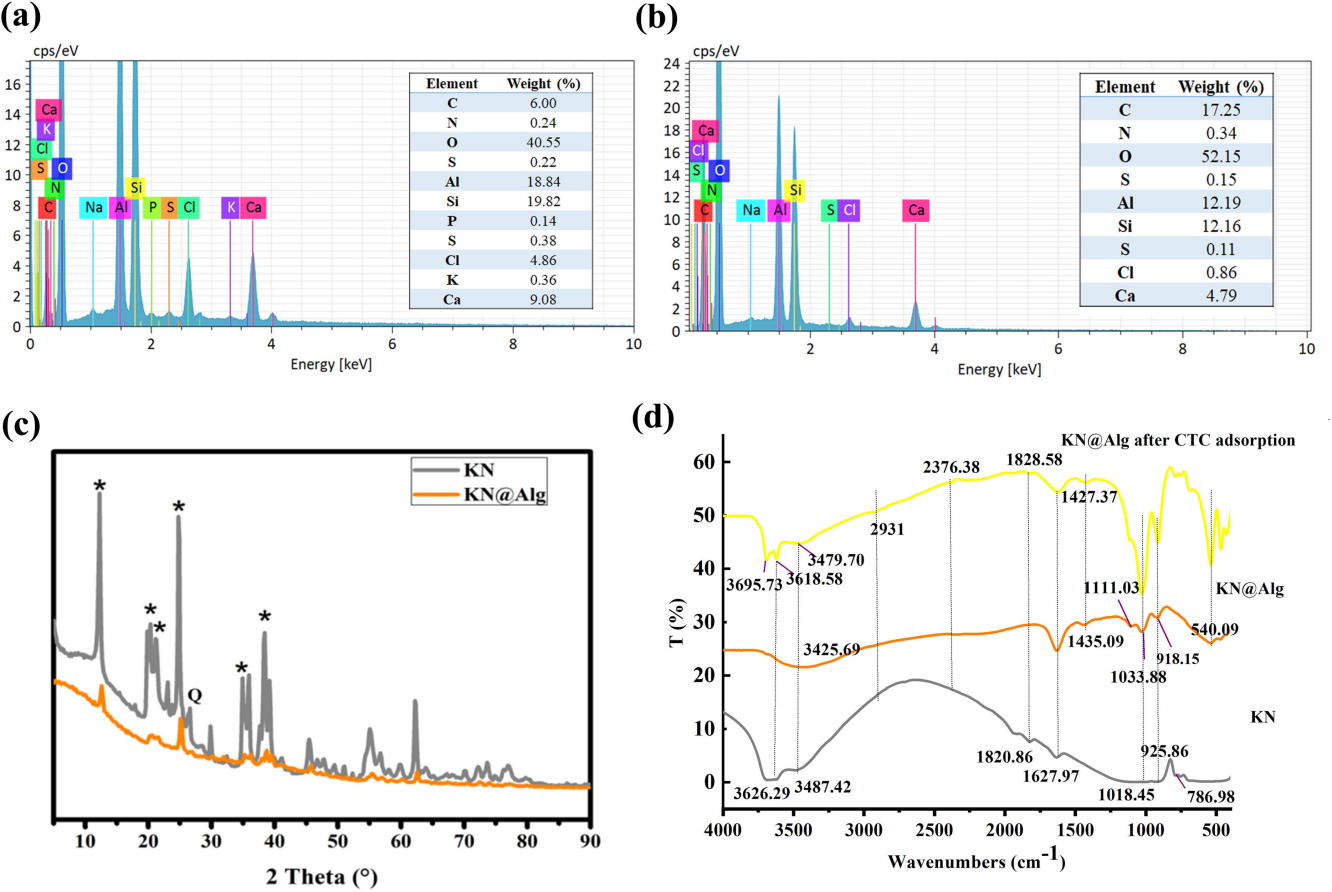
(a) EDS spectra of KN@Alg and (b) after CTC adsorption, (c) XRD patterns of KN and KN@Alg, and (d) FTIR spectra of KN, KN@Alg and KN@Alg after CTC adsorption.

The elemental distribution of the KN@Alg beads and their ability to adsorb CTC were investigated using EDS analysis. A comparison of figure 3a,b revealed an increase in carbon (C) percentage, providing strong evidence for successful CTC adsorption onto the KN@Alg beads. The atomic percentages of calcium (Ca), silicon (Si), and aluminium (Al) in figure 3b were significantly decreased after CTC adsorption compared with those in figure 3a. This difference could be ascribed to the interactions of Ca, Si and Al with CTC during adsorption. These interactions may cause changes in the elemental composition and distribution within the KN@Alg composite beads [28].

Figure 3c shows the XRD patterns of KN and KN@Alg, with key peaks at 2*θ* values of 12.36°, 20.43°, 21.42°, 24.86°, 35.90° and 38.46° [40]. The maximum crystallite size occurred at 24.86°. Peaks for kaolinite were at 12.36° and 24.86°, while quartz shows a peak at 26.6° [41]. The attenuated diffraction peaks observed for KN@Alg compared with pristine KN suggest a homogeneous dispersion of KN within the alginate gel matrix.

FTIR spectra of KN and KN@Alg prior to and after CTC adsorption are displayed in figure 3d. The FTIR analysis of KN revealed characteristic functional groups, with a prominent Si–O stretching vibration at 1018 cm⁻¹ and a spectral band at 786 cm⁻¹ corresponding to Si–O–Al stretching vibrations [42]. The –OH stretching bands were observed at 3626 and 3487 cm^–1^ which were corresponding to the grafting of –OH with Al site of KN clay [43]. FTIR analysis of SA revealed characteristic absorption bands at 1435 cm⁻¹ and 1627 cm⁻¹, corresponding to symmetric and asymmetric vibrations of carboxylate anions, respectively (electronic supplementary material, figure S1) [39,42].

In the FTIR spectrum of KN@Alg, the broad band at 3425 cm^−1^ was attributed to –OH groups of SA. The FTIR spectrum of KN@Alg composite beads retained the characteristic bands of both SA (electronic supplementary material, figure S1) and KN, confirming their successful formation. After CTC adsorption, two distinct vibrational modes related to aluminium hydroxide components were identified in KN@Alg, which were the band at 925 cm^−^¹ representing Al–OH bending vibrations and the sharp absorption at 3695 cm⁻¹ arising from stretching vibrations of hydroxyl groups in the alumina octahedral sheets [42]. Most KN@Alg bands were retained but shifted, indicating CTC interaction. The emergence of a new band at 2931 cm⁻¹, corresponding to C–H stretching vibrations of aliphatic hydrocarbons from CTC, further confirmed successful CTC loading, consistent with previous literature [15].

### 3.2. Adsorption performance of activated carbon, kaolin and KN@Alg

#### 3.2.1. Influence of factors or parameters

**Figure 4.**
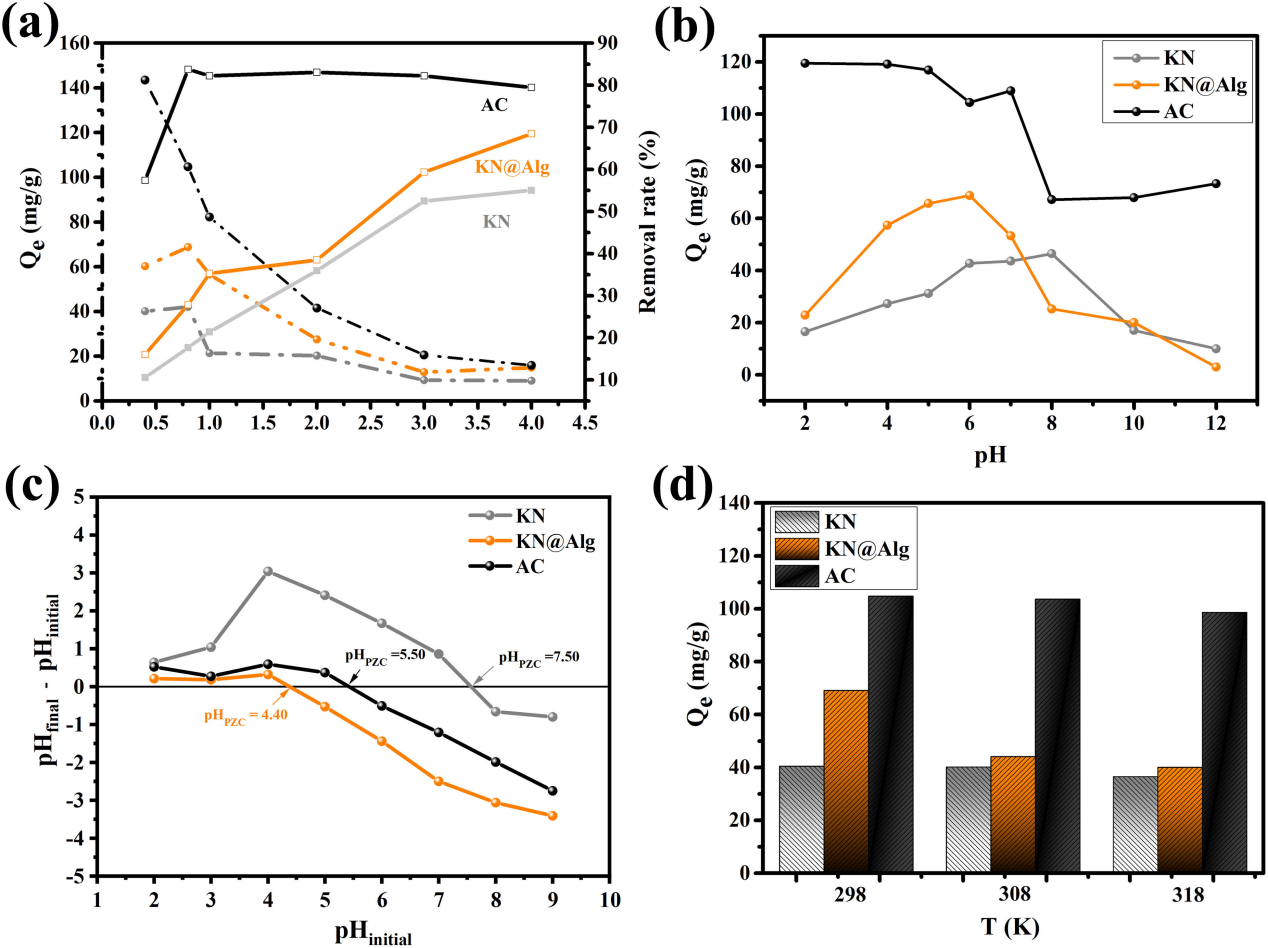
(a) The effect of adsorbent dosage on CTC adsorption capacity and removal rate, (b) effect of initial solution pH value, (c) pH_pzc_ of AC, KN and KN@Alg, and (d) the effect of temperature on CTC adsorption.

Figure 4a shows that the CTC removal efficiency by both KN and KN@Alg increased with adsorbent dosage, reaching maximum values of 55.04% and 68.44%, respectively, at 4 g l^−1^. This observation suggests that increasing the adsorbent dosage enhances CTC removal by providing greater availability of active adsorption sites. The data demonstrate that increasing the KN@Alg dosage from 0.4 to 0.8 g l^−1^ moderately enhanced CTC adsorption capacity due to greater active site availability [44]. However, at dosages above 0.8 g l^−1^, although the increased number of binding sites improved overall removal efficiency, the corresponding decrease in aqueous CTC concentration reduced adsorption capacity per unit mass because of active site underutilization [28,45].

At low adsorbent dosage, AC demonstrated superior CTC removal efficiency (79.45%) compared with KN and KN@Alg composites, owing to its highly porous structure. However, considering the cost of AC, the KN-based composites showed promising removal capacity while offering significant economic advantages, making them viable sustainable alternatives to AC for CTC adsorption.

As shown in figure 4b, maximum CTC adsorption capacities were observed at pH 8 for KN (42.76 mg g^−1^) and pH 6 for KN@Alg (68.74 mg g^−1^), with both materials exhibiting significant capacity reduction at higher pH values. These results align with previous studies [46]. CTC is an amphoteric molecule, the species of which are governed by the solution pH value. When pH < 3.3, 3.3 < pH < 7.6, 7.6 < pH < 9.3 and pH > 9.3, the dominant CTC species were CTC^+^, CTCH^2±^, CTCH^−^, and CTC^2−^, respectively [47]. The pH_pzc_ values of AC, KN and KN@Alg were measured as approximately 5.5, 7.5 and 4.4 (figure 4c), indicating that the adsorbent surface was negatively charged at pH > pH_pzc_, and positively charged at pH < pH_pzc_. The pronounced electrostatic repulsion between CTC^+^ and the similarly charged surfaces of KN and KN@Alg adsorbents significantly hindered CTC adsorption, resulting in low adsorption performance. Conversely, when the pH was maintained between 4 and 7, CTC transitioned into neutral molecular forms and zwitterionic species, minimizing electrostatic repulsion. The CTC^+^ and CTCH₂^±^ species of CTC can interact electrostatically with negatively charged adsorbent (e.g. –X⁻···CTCH₂^±^). This enhanced electrostatic attraction between CTC and either KN or KN@Alg at appropriate pH conditions consequently increased the overall adsorption capacity. Although electrostatic repulsion between CTC^+^ and adsorbent likely occurred at pH < 4, the observed adsorption capacity remained significant (20 mg g^−1^ at pH 2). This suggests that alternative mechanisms—including hydrogen bonding, chelation and pore filling—contributed substantially to CTC adsorption by the clay beads.

The CTC removal efficiency by AC remained relatively constant across the pH range of 2.0 to 7.0 and even at low pH (2−3.3), where the adsorbent and CTC both have the same charge, the adsorbent showed a high removal capacity (119.46 mg g^−1^) which might be explained in that CTC adsorption on AC relied on van der Waals, H−π, and π–π interactions over electrostatic attraction [48].

With pH increasing (pH > 7), CTC molecules underwent complete deprotonation, becoming negatively charged. Electrostatic repulsion occurs when both the adsorbate (CTC) and adsorbent surface carry negative charges, creating unfavourable conditions for adsorption. Hence CTC adsorption onto the adsorbents decreased with increasing pH values. Moreover, this phenomenon may also be attributed to competition between hydroxyl ions (OH⁻) and CTC molecules for active adsorption sites [49,50].

As shown in figure 4d, increasing temperature from 298 K to 318 K reduced CTC adsorption capacity for all adsorbents (KN: 40.42−38.15 mg g^−1^, KN@Alg: 69.08−54.22 mg g^−1^, AC: 104.71−97.83 mg g^−1^), confirming the exothermic nature of adsorption. The pronounced decrease for KN@Alg beads might be attributed to partial destabilization of alginate’s porous matrix at higher temperatures, reducing accessible binding sites [51]. The milder decline for KN and AC suggests their adsorption relies more on temperature-resistant mechanisms like pore filling [52]. Optimal performance at 298 K aligns with reported antibiotic adsorption trends on clay–biopolymer composites, where ambient temperatures balance physisorption and chemisorption [53].

**Figure 5.**
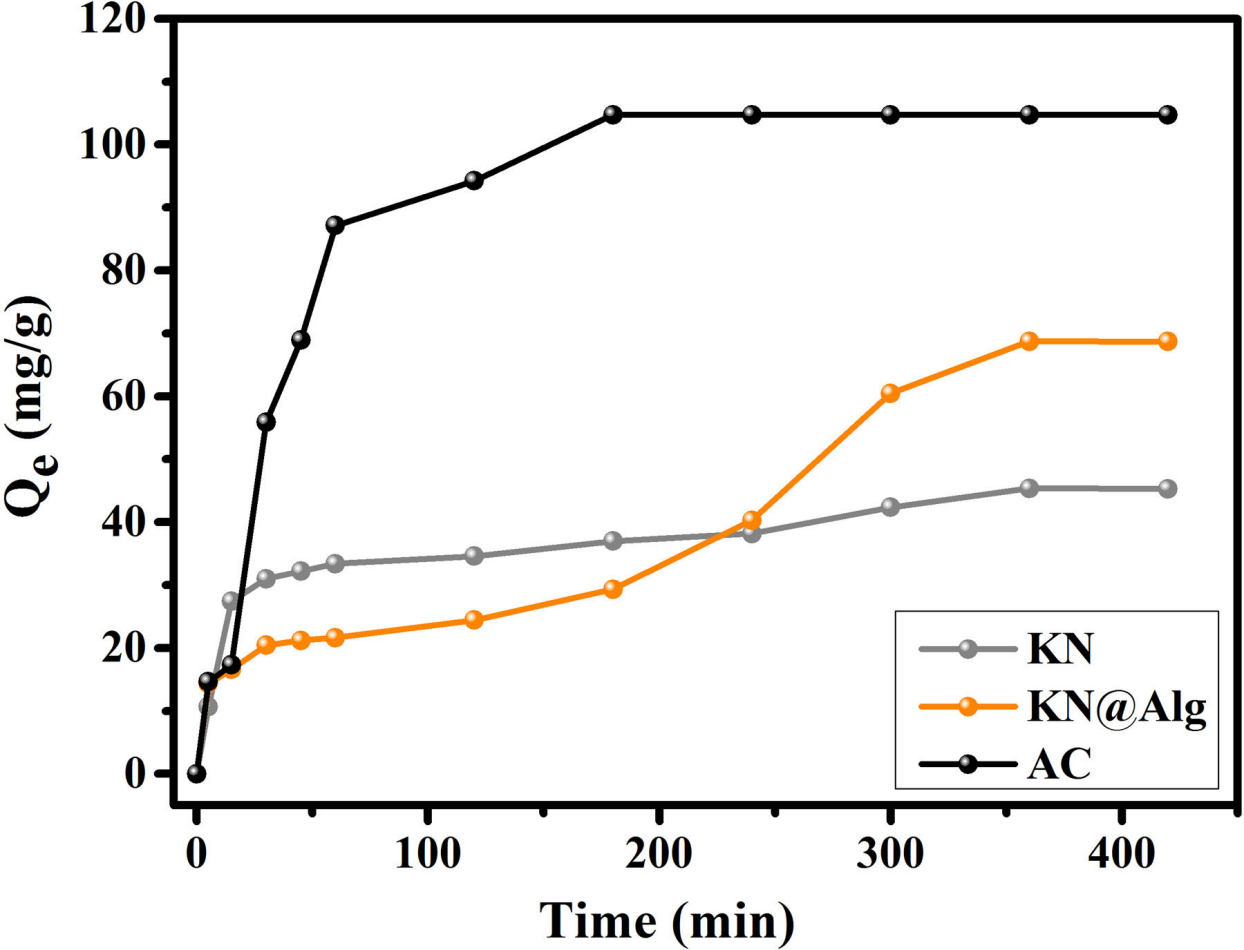
The effect of adsorption time on CTC adsorption onto AC, KN and KN@Alg beads.

As shown in figure 5, during the initial adsorption phase (0−180 min), KN demonstrated both higher and faster CTC uptake compared with KN@Alg beads. Both adsorbents reached an equilibrium capacity of 40 mg g^−1^ by 240 min. Following this initial phase, the CTC adsorption rate on KN decreased progressively until reaching equilibrium at approximately 360 min, with a maximum capacity of 45.30 mg g^−1^. By contrast, KN@Alg continued to adsorb CTC, also reaching equilibrium at about 360 min but with a higher capacity of 68.70 mg g^−1^. Initially, the limited contact time between CTC molecules and the active sites on the beads resulted in low adsorption capacity. As time progressed, the driving force facilitated the transfer of CTC molecules to the KN@Alg surface, enhancing contact and adsorption. This led to intra-granular mass transfer, allowing CTC absorbed on the outer surface to gradually reach the internal active sites through intra-particle diffusion, ultimately saturating those sites. The higher loading capacity of KN@Alg was attributed to the increase in the binding sites of the composite after SA modification. For AC, the adsorption of CTC reached equilibrium faster (180 min) than KN and KN@Alg due to the porous structure of AC which facilitates the rapid diffusion of CTC into the active sites.

#### 3.2.2. Adsorption kinetics studies

**Figure 6.**
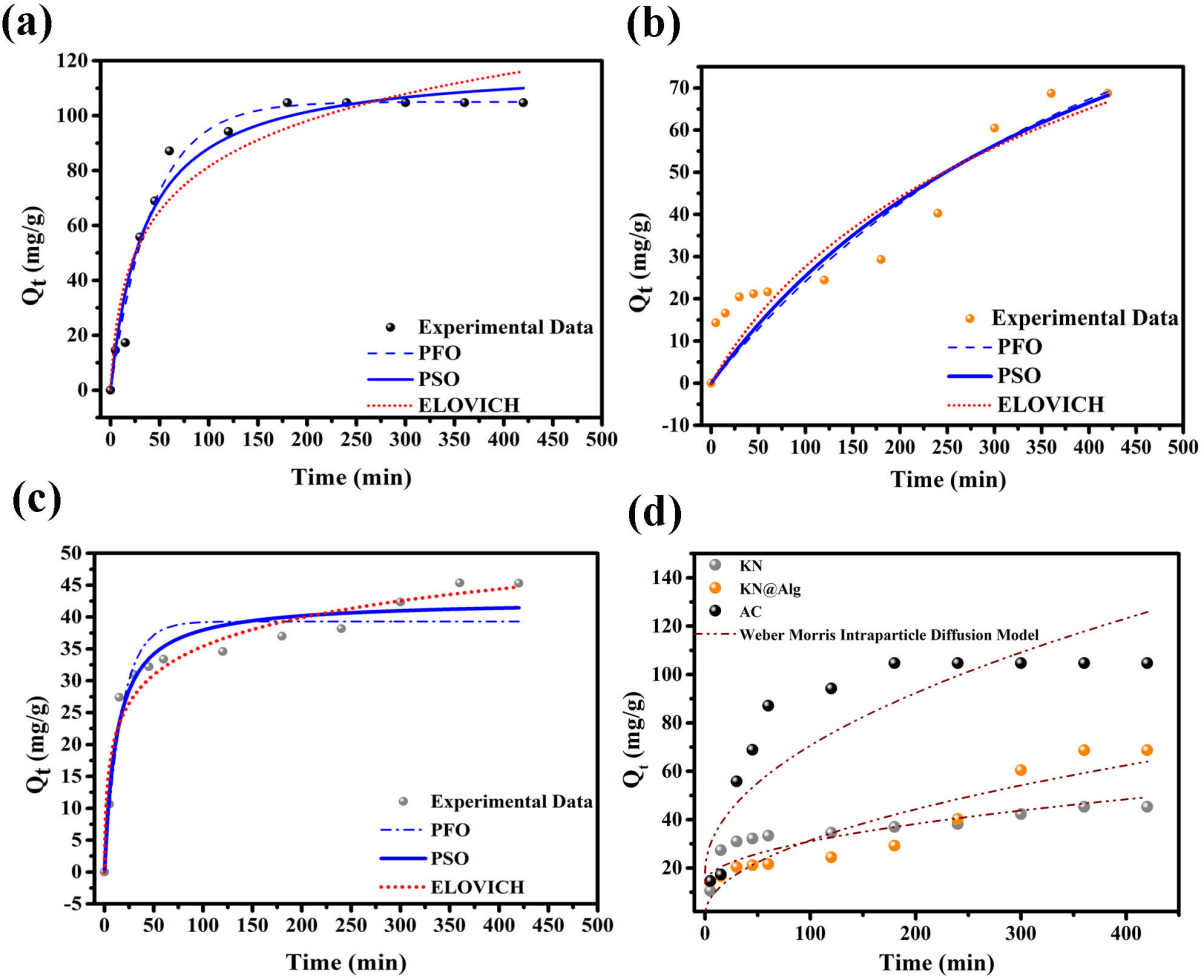
PFO, PSO and Elovich kinetic models for CTC adsorption onto AC (a), KN@Alg (b) and KN (c), and (d) the IPD model for CTC adsorption onto AC, KN and KN@Alg.

Table 1 summarizes the kinetic fitting constants for all three adsorbents with corresponding model fits graphically presented in figure 6. For KN and KN@Alg beads, the PSO model displayed higher *R*² values (0.951 and 0.838, respectively) compared with the PFO model (0.905 and 0.834, respectively), indicating that chemical adsorption (chemisorption) may primarily governed the process. For KN, the *Q_e_* values from the PSO model aligned closely with the experimental results, suggesting a combination of physisorption and chemisorption mechanisms, with chemisorption being dominant [54]. By contrast, for the KN@Alg beads, the *Q_e_* values from the PFO model aligned closely with the experimental results, indicating higher probability of the dominance of physisorption. Interestingly, AC showed a higher *R*² value for the PFO model (0.982) compared with the PSO model (0.965), further supporting the predominance of physisorption (*Q*_cal_ ≅ *Q*_exp_) [55]. The Elovich model was used to explain the chemisorption process for KN and KN@Alg, with good correlation coefficients (*R*²) indicating that adsorption may be controlled by chemisorption at heterogeneous binding sites, with little impact from desorption or interactions at low surface coverage [56].

**Table 1.** Kinetic model simulation parameters for CTC adsorption by the studied adsorbents with initial CTC concentration at 100 mg l^−1^.

kinetic models	parameters	AC	KN	KN@Alg
PFO	*Q_e_*_(exp)_ (mg g^−1^)	104.720	45.300	68.700
	*Q_e_*_(cal)_ (mg g^−1^)	105.029	39.304	70.616
	*K*_1_ (min^−1^）	0.023	0.058	0.002
	*R* ^2^	0.982	0.905	0.834
PSO	*Q_e_*_(cal)_ (mg g^−1^)	119.268	42.668	144.307
	*K*_2_ (g mg min^−1^）	2.374 × 10^−4^	0.001	1.481 × 10^−5^
	*R* ^2^	0.965	0.951	0.838
Elovich	*α* (mg g min^−1^)	6.486	15.107	0.392
	*β* (g mg^−1^)	0.040	0.153	0.024
	*R* ^2^	0.923	0.952	0.846
IPD	*K*_id_ (mg (g min^1/2^))	5.262	1.738	3.101
	*C_i_* (mg g^−1^)	18.003	13.678	0.466
	*R* ^2^	0.790	0.751	0.888

The IPD model (figure 6d, table 1) was further employed to elucidate the adsorption mechanism and identify rate-controlling steps in the process. Generally, when the *Q_t_* versus *t*^1/2^ plot exhibits linearity passing through the origin, this signifies intra-particle diffusion as the predominant control mechanism. Conversely, an intercept deviation from the origin implies additional rate-limiting factors beyond intra-particle transport [10]. These results indicated that the CTC adsorption on the studied sorbents involved a multi-step process, including instantaneous surface adsorption, intermediate inter-particle diffusion through boundary layers, and final intra-particle diffusion into the porous matrix. Similar trend was observed in our previous researches [28].

#### 3.2.3. Adsorption isotherms studies

**Figure 7.**
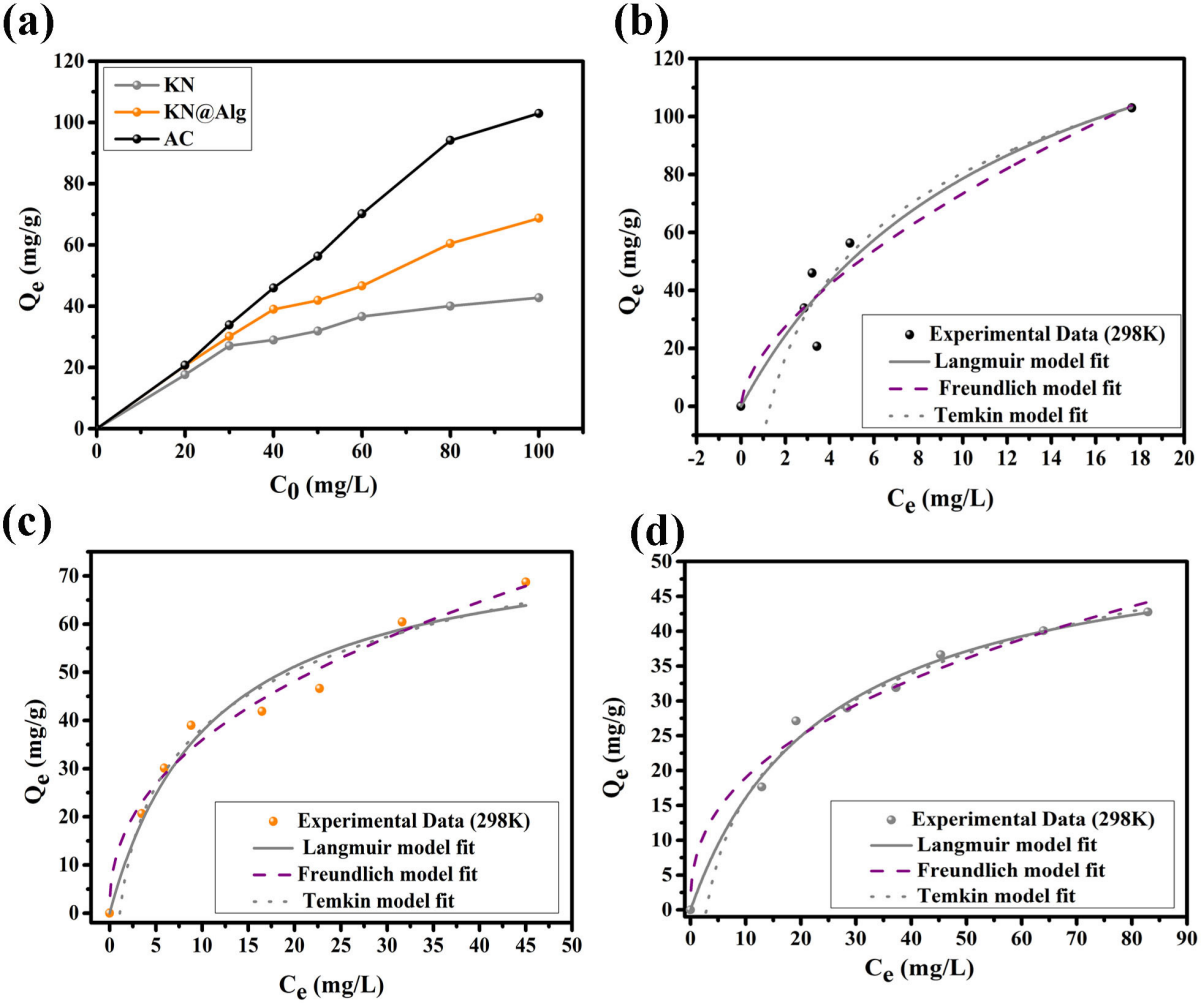
(a) The influence of initial solution concentration on CTC adsorption by AC, KN and KN@Alg. The fitting of Langmuir, Freundlich and Temkin isotherms on CTC adsorption by AC (b), KN@Alg (c) and KN (d).

Figure 7a displays that the adsorption capacity was influenced by CTC concentration, which increased with rising CTC concentration. The adsorption capacities for AC, KN and KN@Alg were measured at 102.96 mg g^−1^, 42.76 mg g^−1^ and 68.74 mg g^−1^, respectively. This result may be attributed to the substantial initial CTC concentration, which serves as a potent driving force for the migration of CTC from the liquid phase to the solid phase within the adsorption system [57].

The nonlinear fitting outcomes for the isotherm models are presented in figure 7b–d, with parameter values detailed in table 2. Based on the Langmuir model, the adsorption process occurs on a homogeneous surface, and the adsorbate forms a monolayer [58]. The Langmuir model displayed high coefficient (*R*^2^) of 0.911, 0.988 and 0.955 for AC, KN and KN@Alg adsorbents. The sorption intensity *R*_L_ ranged from 0.111−0.383, 0.549−0.196 and 0.357−0.101 for AC, KN and KN@Alg, respectively, and suggested that CTC adsorption by the studied adsorbents was favourable [33]. The theoretical maximum capacities of AC, KN and KN@Alg calculated by Langmuir reached 176.151, 55.178 and 79.679 mg g^−1^, respectively (table 2).

**Table 2.** Isotherm model parameters for CTC adsorption onto AC, KN and KN@Alg beads.

isotherm models	parameters	adsorbents		
		AC	KN	KN@Alg
Langmuir	*Q*_e(exp)_ (mg g^−1^)	102.960	42.7570	68.740
	*Q*_max_ (mg g^−1^)	176.151	55.178	79.679
	*K*_L_ (l mg^−1^)	0.080	0.041	0.089
	*R*_L_	0.111–0.383	0.549–0.196	0.357–0.101
	*R* ^2^	0.911	0.98835	0.955
Freundlich	1/*n*_F_	0.609	0.3995	0.423
	*K*_F_ (mg g^−1^)/(l mg^−1^)^1/*n*^	18.051	7.56723	13.556
	*R* ^2^	0.841	0.93595	0.956
Temkin	*K*_T_	0.764	0.34762	0.908
	*α* (*RT*/*b*_T_)	39.612	12.88596	17.358
	*b*_T_	62.519	192.265	142.73
	*R* ^2^	0.911	0.988	0.9674

The Freundlich isotherm model involves an empirical equation describing multilayer adsorption on heterogeneous surfaces with energetically distinct binding sites. The Freundlich model exhibited lower correlation coefficients (*R*² = 0.841 for AC; 0.935 for KN) compared with the Langmuir model. However, KN@Alg showed a higher *R*^2^ of 0.95 for both models which implied that the adsorption process fitted well the Langmuir and Freundlich models (table 2). The ratio 1/*n*_F_ offers surface heterogeneity information [59]. The 1/*n*_F_ value was 0.399 for KN, 0.423 for KN@Alg and 0.609 for AC signifying that KN and its composites presented high heterogeneity degree.

The Temkin model essentially describes a linear decrease in adsorption heat of adsorbent with increasing coverages, reflecting the interactions between the adsorbent and adsorbate [60]. This model exhibited the highest *R*² values, suggesting it provided the best fit to the experimental adsorption data (table 2). The Temkin constant, *b*_T_, represented the variation in adsorption energy, with values of 62.519, 192.265 and 142.73 for AC, KN and KN@Alg, respectively. These values suggested that CTC adsorption onto the adsorbents might occur exothermically within the studied concentration range [60]. It can be concluded that the adsorption of CTC onto AC and KN occurs on a homogeneous surface, whereas adsorption onto KN@Alg may take place on both homogeneous and heterogeneous surfaces.

#### 3.2.4. Thermodynamic studies

**Figure 8.**
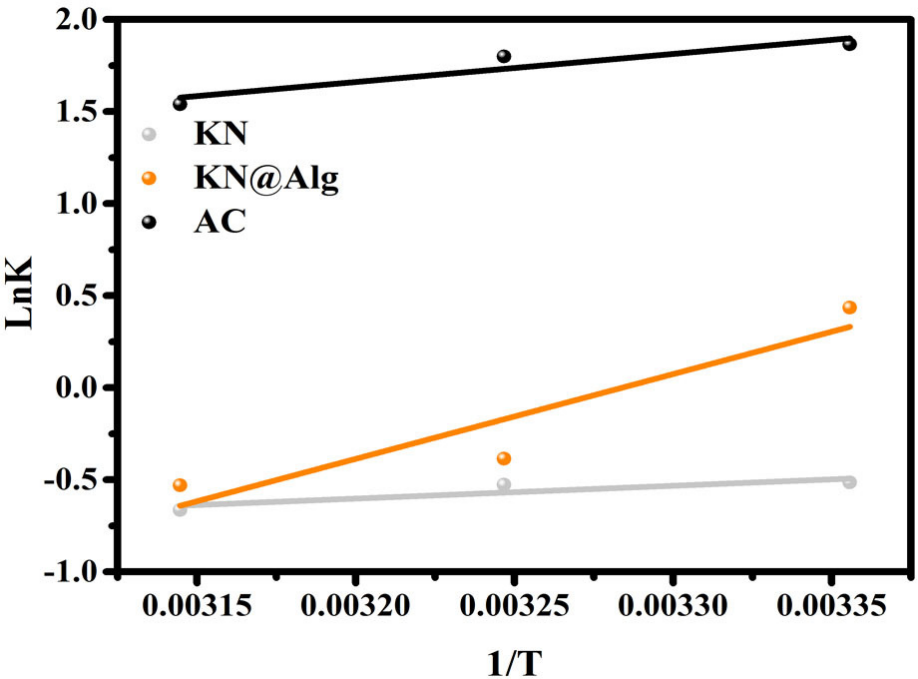
Plot of ln *K* versus 1/*T*.

The thermodynamic parameters Δ*H°* (J mol^−1^) and Δ*S°* (J (K mol)^−1^) for CTC adsorption onto AC, KN and KN@Alg were determined from the slope and intercept of the linear regression of ln *K* versus 1/*T* (figure 8) and are summarized in table 3. The negative enthalpy change (Δ*H° *< 0) confirmed the exothermic nature of CTC adsorption onto the adsorbents. Therefore, a decrease in temperature favoured the adsorption process. Δ*S°* was related to the sorption stability, and its negative values revealed the decrease in randomness and free energy between the solid–liquid interface, resulting in stable adsorption. The Δ*G°* of AC was negative at all three temperatures, demonstrating that CTC adsorption into AC was spontaneous. For KN@Alg, the Δ*G°* was negative at only 298 K, implying that the KN@Alg adsorption of CTC was spontaneous and feasible at low temperature. However, Δ*G°* values were positive at all temperatures, suggesting that CTC adsorption onto KN was less favoured and difficult. Thus, the encapsulation of KN into Alg hydrogel enhanced the adsorption properties of KN. Physisorption is defined by an absolute value of enthalpy between 5 and 20 kJ mol^−1^. Whereas that of chemisorption has much larger enthalpies in the range of chemical bonding. The calculated Δ*H°* values for CTC adsorption on AC and KN were between 5 and 13 kJ mol^−1^ which validated that CTC adsorbed on AC was via physisorption. The calculated Δ*H°* obtained for CTC adsorption on KN@Alg (38.267 kJ mol^−1^), clearly, was indicative of chemisorption process. These results are consistent with the conclusions drawn from the kinetic studies.

**Table 3.** The thermodynamic parameters.

	*T* (K)	Δ*G°* (J mol^−1^)	Δ*H°* (J mol^−1^)	Δ*S°* (J (K mol^−1^))
AC	298	−4697.150	−12681.060	−26.791
	308	−4429.296		
	318	−4161.382		
KN	298	1222.700	−5790.4080	−23.533
	308	1458.039		
	318	1693.378		
KN@Alg	298	−824.684	−38267.550	−125.647
	308	431.78		
	318	1688.25		

### 3.3. Regeneration studies

**Figure 9.**
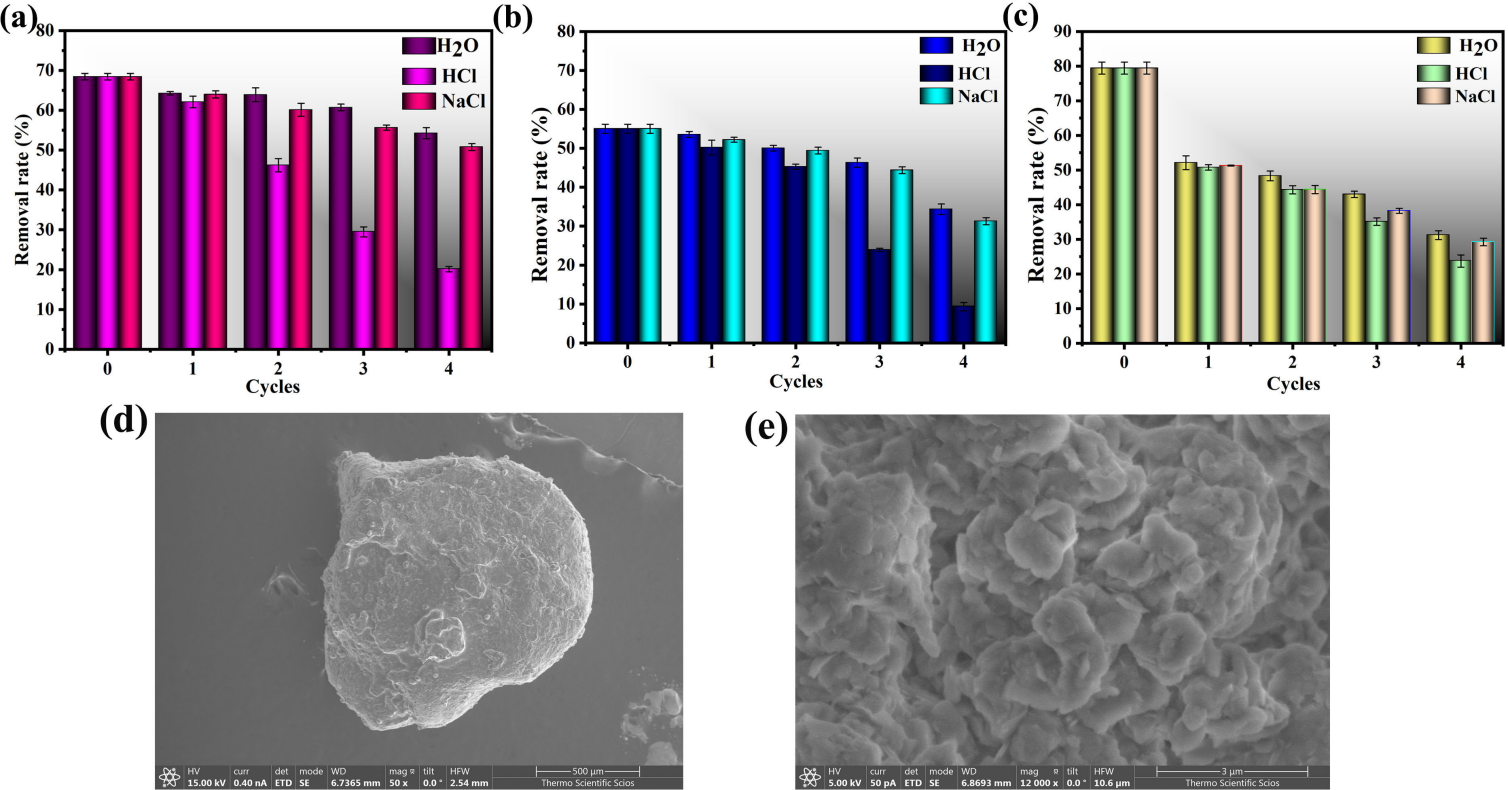
(a) Reusability studies of KN@Alg, (b) KN and (c) AC by different eluents. SEM images of KN@Alg after four regeneration cycles (d,e).

Regeneration studies demonstrated that distilled water (H_2_O) was the most suitable eluent for CTC desorption (figure 9). After four regeneration cycles, KN@Alg maintained stable adsorption performance, exhibiting only a 14.19% reduction in CTC removal efficiency. This minimal efficiency loss, coupled with consistent regeneration behaviour, confirms both the effective restoration of active binding sites and the material’s excellent reusability potential. Conversely, the removal capacity of KN reduced by 20.67% and that of AC significantly reduced by 48.24% compared with KN@Alg. This indicated that Alg improved the regeneration performance of KN. Regeneration studies showed that KN@Alg could be successfully regenerated for at least four cycles, and it was efficiently practical on a large scale.

The SEM micrographs in figure 9d,e demonstrate that the KN@Alg beads maintained their structural integrity and microporous network after four regeneration cycles, confirming their stability as reusable adsorbents. This remarkable recyclability suggests that KN@Alg beads are promising for multiple treatment cycles of CTC-contaminated wastewater, offering significant cost advantages compared with single-use adsorbents.

### 3.4. Practical application of KN@Alg beads

#### 3.4.1. Solid-phase extraction cartridge experiment

**Figure 10.**
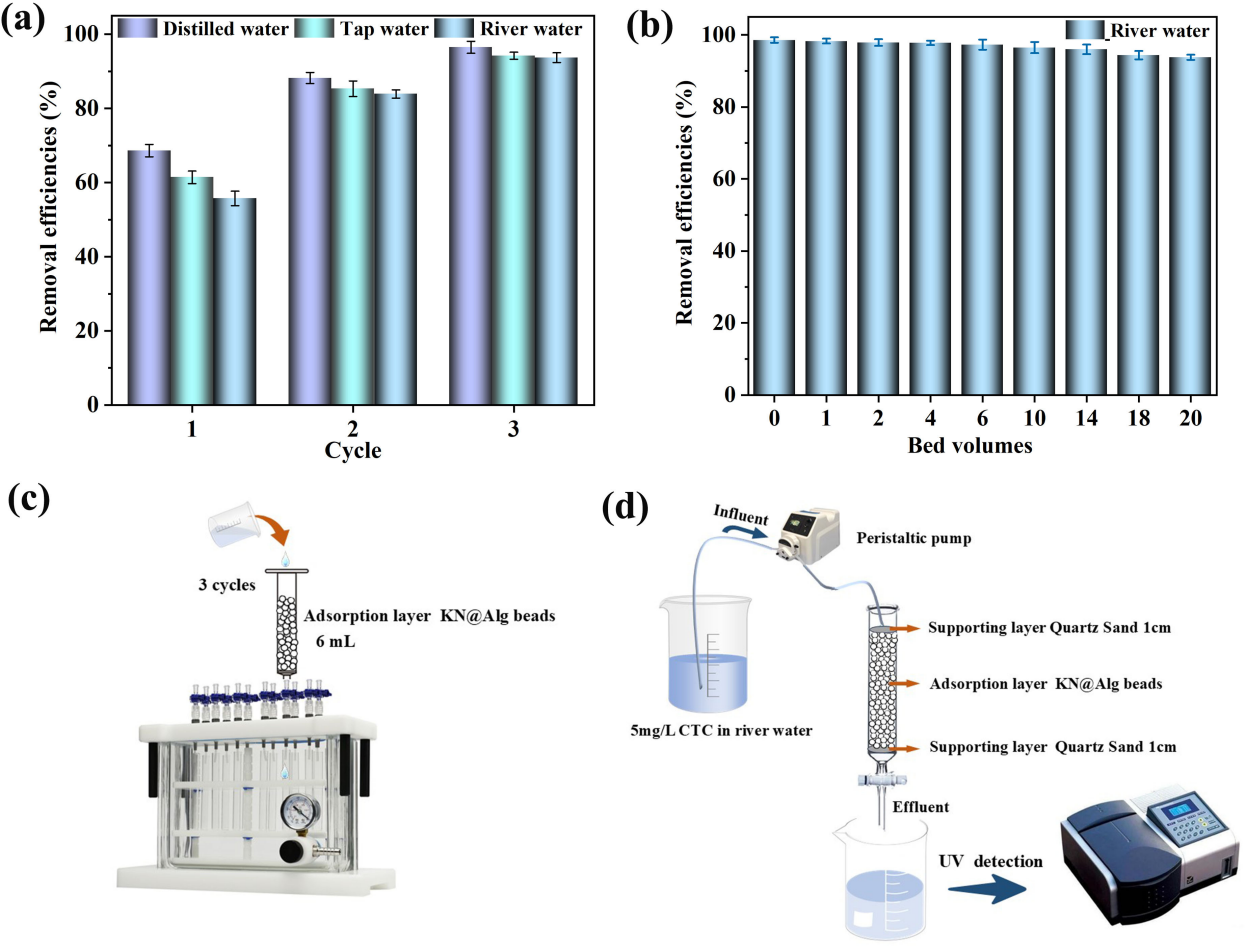
(a) The removal efficiencies of CTC adsorption by SPE cartridge and (b) the continuous bead column. (c) Schematic diagram of the SPE cartridge and (d) continuous bead column.

As displayed in figure 10a, when the initial CTC concentration of the solution was 5 mg l^−1^, after one elution treatment with KN@Alg beads, the CTC concentrations in distilled water, tap water and river water eluates decreased to 68.6%, 61.4% and 55.7% of the initial value, respectively. After three elution cycles, the removal efficiencies of the syringe unit reached approximately 96.5%, 94.2% and 93.7% for these three types of water, respectively.

#### 3.4.2. Continuous bead column experiment

To systematically evaluate the practical application potential of KN@Alg beads, this study conducted dynamic removal experiments of CTC from river water using an upgraded continuous-flow glass bead column adsorption system. Throughout the 20-bed volumes continuous operation, the KN@Alg bead column system maintained a high CTC removal efficiency of 93.6% (figure 10b). Notably, this composite system demonstrated remarkable engineering advantages. Firstly, the biocompatibility of SA matrix and natural KN aligns with the design requirements for environmentally friendly water treatment agents. Furthermore, the material retained considerable adsorption capacity after four consecutive regeneration cycles using deionized water, confirming its excellent reusability and stability. These findings collectively demonstrated that the KN@Alg bead column system holds significant promise for industrial-scale applications in wastewater treatment, particularly for advanced removal of antibiotic contaminants.

### 3.5. Adsorption cost of KN@Alg beads

The cost associated with removing CTC using KN@Alg beads was evaluated, as presented in equation (3.1) [61]:


(3.1) $${\text{Adsorption cost (US\$ g}}^{-1})=\frac{{\text{Cost of adsorbent (US\$ g}}^{-1})}{{\text{Adsorption capacity (mg g}}^{-1})\times 10^{-3}\;\left({g\;mg}^{-1}\right)}.$$

The adsorbent cost assessment incorporated the expenses of chemicals and energy consumption during KN@Alg preparation. The relevant chemical costs were: SA (8.36 US$ kg^−1^), KN (3.90 US$ kg^−1^) and CaCl₂ (10.59 US$ kg^−1^). The energy cost for synthesizing KN@Alg was estimated based on an electricity price of 0.0175 US$ g^−1^. Assuming the adsorbent can be reused for four cycles, the calculated cost for removing a unit weight of CTC was 0.095 US$ g^−1^, indicating that KN@Alg is a cheap adsorbent. In comparison, the corresponding cost for an AC (21.16 US$ kg^−1^) adsorbent was calculated to be 0.15 US$ g^−1^. Therefore, KN@Alg proves to be an economically viable and reusable material for wastewater treatment, offering substantial reductions in material production expenses compared with commercial adsorbents [61].

### 3.6. Interaction mechanism of chlortetracycline onto KN@Alg based on X-ray photoelectron spectroscopy analysis

**Figure 11.**
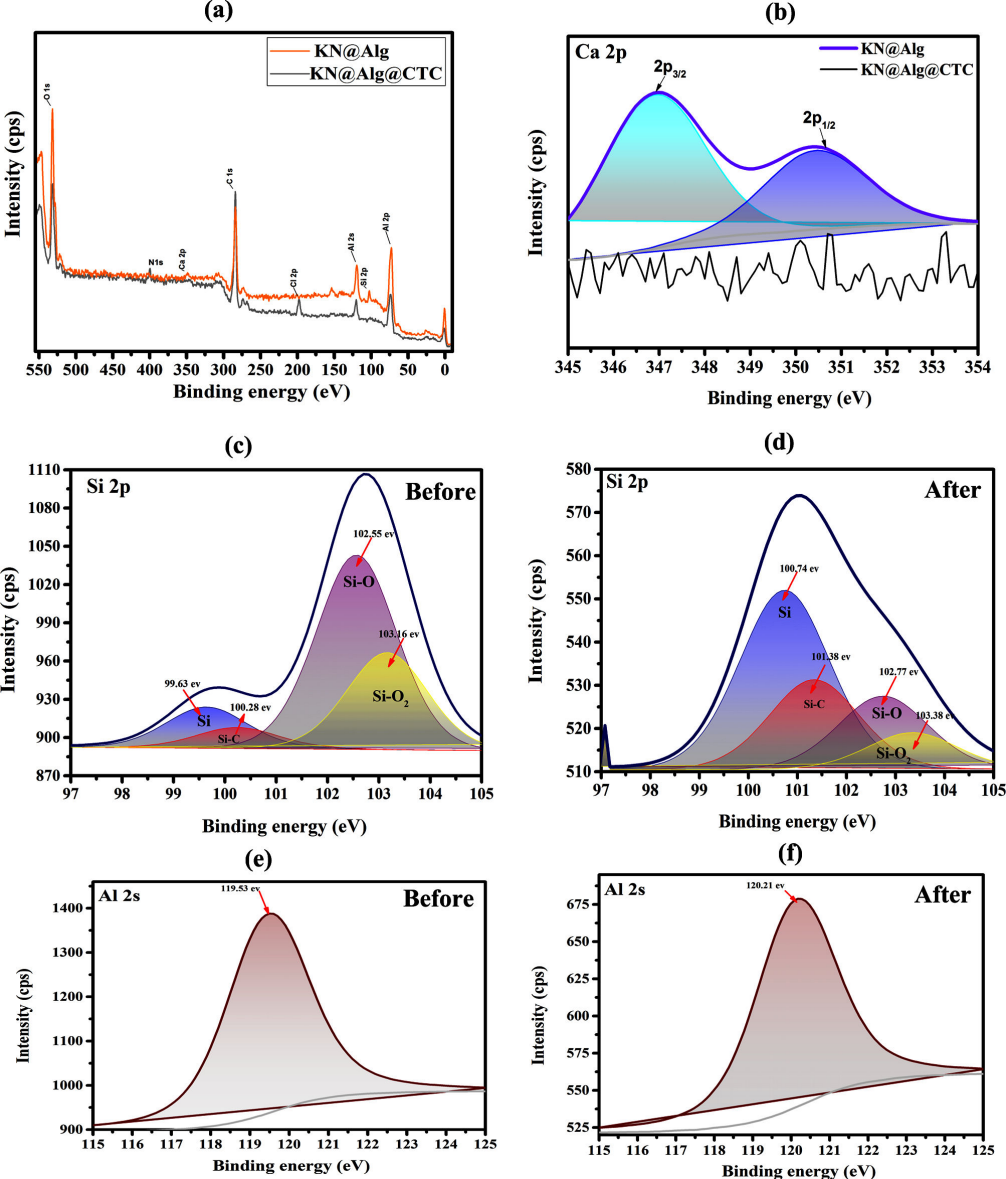
(a) XPS wide-scan spectra, (b) Ca 2p spectra, (c,d) Si 2p spectra, (e,f) Al 2p spectra before and after CTC adsorption.

Figure 11 depicts the high-resolution XPS spectra of KN@Alg beads, both prior to and after adsorption. The survey XPS spectra (figure 11a) indicated that the primary elements of KN@Alg were C, N, O, Al, Si and Ca. After the adsorption of CTC, a characteristic peak emerged near 400 eV in the XPS spectrum of the composite beads (figure 11a), which could be assigned to the N 1s peak [62]. This observation suggests that CTC was effectively immobilized on the surface of the KN@Alg beads. The peak intensity of C also increased after CTC adsorption, further indicating the adsorption process [63]. To explore the adsorption mechanism of CTC by the prepared beads, the changes in Ca, Al and Si elements before and after CTC adsorption were investigated. The results displayed that following the adsorption of CTC, the characteristic peak corresponding to Ca 2p disappeared (figure 11b), indicating that hydroxyl (–OH) groups and a keto group (C=O) in CTC might interact chemically with calcium ions, potentially forming a complex or precipitate. This interaction may alter the electronic environment of the calcium, leading to the disappearance of its XPS peak. Moreover, the hydroxyl, carbonyl and amino groups present within the CTC molecules donated a substantial quantity of electrons to Ca, facilitating the creation of metal complexes with Ca²^+^ via cation–π and cation–n bonding interactions [63,64]. Another possible mechanism leading to the disappearance of the Ca peak is the cation exchange mechanism between Ca²^+^ and CTC. This exchange has been identified as a key mechanism in the interaction between clay-based adsorbents and tetracyclines. Evidence supporting this includes the observation of desorbed metal cations, including Ca²^+^, which originate from clays or clay–alginate composites [25].

In figure 11c,d, the characteristic peaks of Si 2p at 99.63, 102.55 and 103.16 eV, which correspond to Si, Si–O and Si–O_2_ groups, are shifting to 100.74, 102.77 and 103.38 eV, suggesting that these groups are important in the CTC adsorption process, engaging in electrostatic interactions between Si–O^−^ and CTC^+^, forming hydrogen bonds between Si–O/Si–O_2_ and hydroxyl groups of CTC, and participating in cation–n (like surface complexation) and cation–π interactions of Si^4+^ with CTC. Additionally, as shown in figure 11e,f, the characteristic Al 2p peak shifted by approximately 0.68 eV after CTC adsorption, suggesting that Al might also participate in complex formation with CTC, similar to the behaviour observed for Si [20,25]. These outcomes were consistent with EDS analysis results.

**Figure 12.**
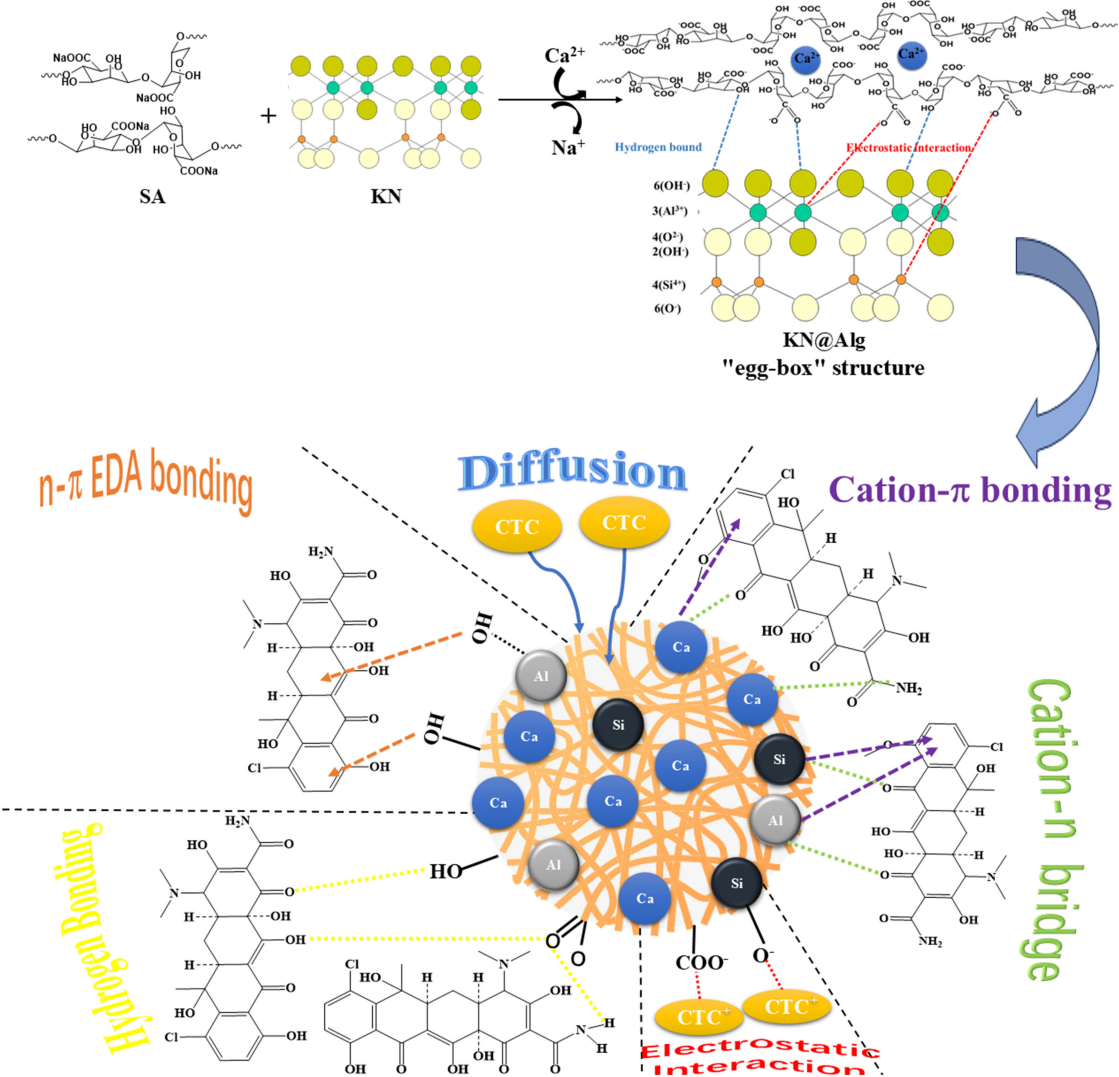
Proposed interaction mechanism of CTC on KN@Alg beads.

The possible adsorption mechanism of CTC in this work is illustrated in figure 12. Based on the adsorbent characterization results, pH studies, kinetics and isotherm data of CTC adsorption by the adsorbent, it was clear that CTC adsorption onto KN@Alg was influenced by chemisorption, physisorption and the surface heterogeneity of the KN@Alg beads. Moreover, calcium in calcium-crosslinked SA, KN encapsulated in the beads, and calcium bound to KN minerals were the three potential adsorption sites in calcium-crosslinked KN@Alg beads. The mechanism for CTC removal might involve strong n–π EDA interactions and hydrogen bonding between the active oxygen-containing functional groups on KN@Alg and CTC molecules. Additionally, the cation–n and cation–π bonding bridges between CTC and Ca crosslinked KN@Alg, along with hydrogen bonding, electrostatic interactions, cation exchange and CTC diffusion into the pores of the beads, all contribute to the adsorption process.

## 4. Conclusion

This study successfully demonstrates the efficacy of kaolin–alginate composite beads (KN@Alg) as a sustainable and cost-effective adsorbent for CTC removal from aqueous solutions. Comprehensive characterization confirmed the successful synthesis of the KN@Alg composite. Key findings highlight the significant advantages of KN@Alg over pristine KN. Regarding adsorption capacity for CTC, KN@Alg (68.74 mg g^−1^) outperformed KN (42.76 mg g^−1^) and approached the capacity of AC (102.96 mg g^−1^). In removal efficiency, both KN@Alg (68.44%) and AC (79.45%) demonstrated superior performance to KN (55.04%). Kinetic analyses indicated rapid adsorption involving both physisorption and chemisorption mechanisms. Isotherm studies revealed homogeneous and heterogeneous adsorption processes. Thermodynamic evaluations confirmed the adsorption as exothermic and spontaneous, particularly favourable at lower temperatures. Additionally, mechanistic studies through XPS analysis identified multiple interaction types including n–π, cation–n, cation–π interactions, cation exchange, hydrogen bonding, electrostatic interactions and pore filling. Critically, KN@Alg beads demonstrated excellent practical adaptability, successfully functioning in both SPE cartridges and continuous bead columns to effectively remove CTC from real water samples. Overall, the composite demonstrated enhanced adsorption performance and regeneration capability compared with unmodified KN. This work presents KN@Alg as an economical, accessible and recyclable adsorbent capable of replacing AC for large-scale water treatment applications targeting CTC antibiotics. The findings provide an efficient and sustainable solution for controlling CTC pollution in aquatic environments.

## Data Availability

The data can be accessed online [65]. Electronic supplementary material is available online [66].
